# Where You Live May Make You Old: The Association between Perceived Poor Neighborhood Quality and Leukocyte Telomere Length

**DOI:** 10.1371/journal.pone.0128460

**Published:** 2015-06-17

**Authors:** Mijung Park, Josine E. Verhoeven, Pim Cuijpers, Charles F. Reynolds III, Brenda W. J. H. Penninx

**Affiliations:** 1 Department of Health and Community Systems, University of Pittsburgh, Pittsburgh, Pennsylvania, United States of America; 2 Department of Psychiatry and EMGO Institute for Health and Care Research, VU University Medical Center, Amsterdam, The Netherlands; 3 Department of Clinical Psychology and EMGO Institute for Health and Care Research, VU University, Amsterdam, The Netherlands; 4 Department of Psychiatry, University of Pittsburgh, Pittsburgh, Pennsylvania, United States of America; University of Newcastle, UNITED KINGDOM

## Abstract

**Background:**

Strong evidence supports that living in disadvantaged neighborhoods has direct unfavorable impact on mental and physical health. However, whether it also has direct impact on cellular health is largely unknown. Thus we examined whether neighborhood quality was associated with leukocyte telomere length, an indicator of cellular aging.

**Methods:**

In May 2014, we extracted and analyzed baseline data from the Netherlands Study of Depression and Anxiety (NESDA), a large epidemiological study of individuals age between 18–65 years (n=2902). Telomere length was determined using quantitative polymerase chain reaction. Neighborhood quality was assessed using modified measures of perceived neighborhood disorder, fear of crime, and noise. We used multivariable linear regression models to examine association between perceived neighborhood quality and telomere length with comprehensive adjustment for individual and community characteristics related to socioeconomic and demographic status, urbanization level, mental and physical health, and lifestyle.

**Results:**

Compared to individuals who reported good neighborhood quality, the mean telomere length of those who reported moderate neighborhood quality was approximately 69 base pair shorter (**β** =-69.33, 95% CI: -119.49, -19.17, p= 0.007), and that of those who reported poor neighborhood quality were 174 base pair shorter (**β** =-173.80, 95% CI: -298.80, -49.01, p=0.006). For illustrative purposes, one could extrapolate these outcomes to 8.7 and 11.9 years in chronological age, respectively.

**Conclusion:**

We have established an association between perceived neighborhood quality and cellular aging over and above a range of individual attributes. Biological aging processes may be impacted by socioeconomic milieu.

## Introduction

Disadvantaged living situations are harmful to health and shorten life expectancy. Evidence from numerous studies and meta-analyses confirms that individuals living in poor and underprivileged neighborhoods experience increased morbidity [1–3], disability [4–8], and mortality [4]. What is largely unknown, however, is whether living in a disadvantaged neighborhood has health impacts on a cellular level.

Leukocyte telomere is nucleic acid-protein complexes at the ends of DNA. Telomere length (TL hereafter) is a biomarker for cellular aging and an indicator of cumulative biological stress [9]. Shorter TL has been associated with mortality [10] and increased risk for a range of morbidities such as cancer [11], heart disease [12], depressive disorders [13], and anxiety disorders [14]. An emerging body of literature has identified demographic, socioeconomic, lifestyle, clinical and environmental factors associated with TL shortening. While TL is shortened continually as we age, male than female and Caucasian race than other races tend to have shorter TL [15–17]. A study showed that widowed, divorced, separated or never married individuals had shorter TL than their married or partnered counterparts [18]. Although inconclusive, existing data suggests that lower educational attainment and lower income level are associated with shortened TL [16]. Unhealthy lifestyles, such as smoking [19, 20], drinking [21] and lower energy expenditure [22, 23] are also associated with TL shortening. Some clinical factors, such as greater Body Mass Index [19] and psychological stress [24] (e.g., early life exposure to trauma [25], and experience of racism [26]), have been linked with shortened TL. A recent study also found that individuals living in areas where less green spaces had shorter TL than their counterparts [27]

Understanding relations between the neighborhood quality and TL is novel and important for several reasons. First, although strong body of literature supports the negative health consequences of disadvantaged living situations, its underlying biological mechanism has not been fully examined. Moreover, the majority of research about health outcomes among individuals living in disadvantaged neighborhood is largely grounded on the psychological stress framework or has had relatively narrow focus specific to stress hormones [4]. Although such research has made a tremendous contribution to our understanding, it has limited power to explain if and how social circumstances intersect with biological processes. Second, existing studies on cellular aging have exclusively concentrated on factors internal to the individual, such as mental and physical disease status or life experience [4]. Thus, identifying factors external to the individual that are associated with telomere lengths may help us to understand the complex interplay of cellular aging and health. Lately, researchers have examined the relationship between cellular aging and social characteristics measured at the level of the individual (e.g., socioeconomic status such as education level and income), yet the results have been inconclusive [28]. Few studies, however, have examined the association between cellular aging and social characteristics measured at the level of neighborhood.

The purpose of this study was to examine if perceived neighborhood quality is associated with TL. Our primary hypothesis was that poor perceived neighborhood quality would be associated with shortened TL. Our secondary hypothesis was that each sub-domain of perceived neighborhood quality (i.e., perceived neighborhood disorders, fear of crime, and noise) would be associated with TL.

## Theoretical Framework

**Fig 1:**
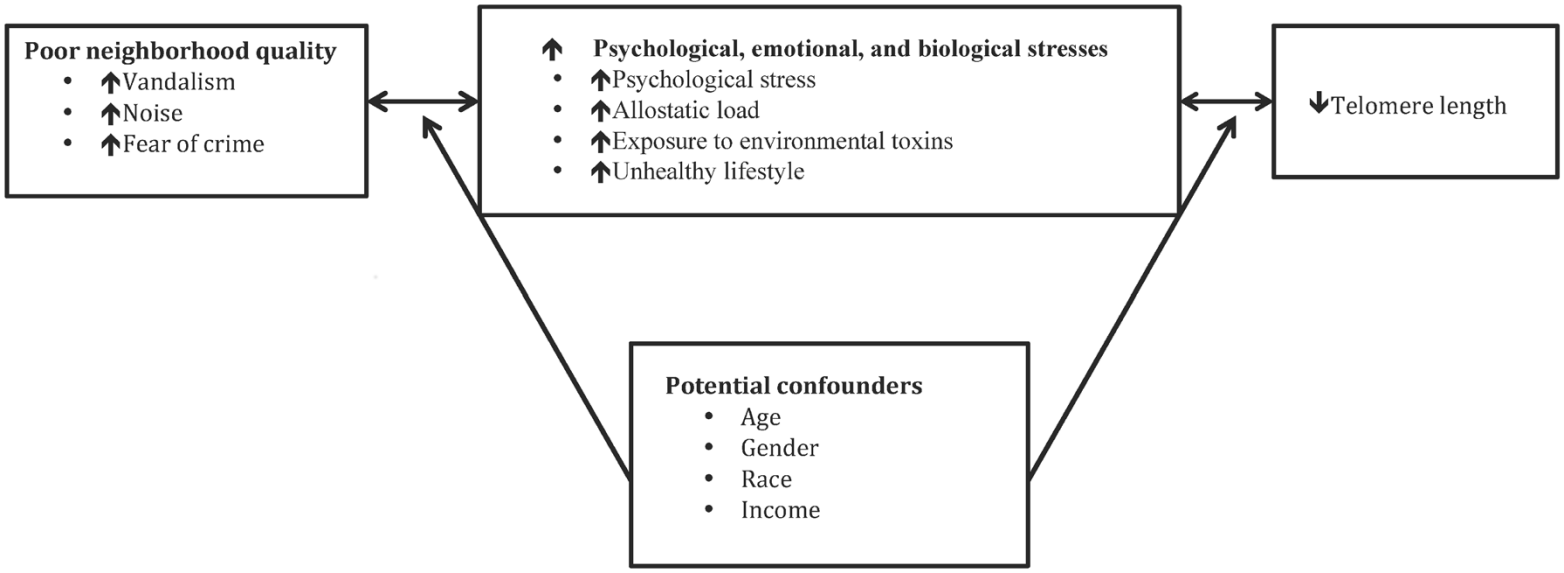
Hypothesized Conceptual Framework.

Our hypotheses were guided by the neighborhood disorder model [29, 30] and a biological framework [31–33]. Fig 1 describes our hypothesized conceptual model of potential pathways linking poor neighborhood quality and shortened TL. Disadvantaged neighborhoods are characterized by high level of neighborhood disorders (e.g. violent crime and vandalism), increased risk for toxic exposure (e.g., noise), and limited access to resources and economic and educational opportunities. Consequently, residents of such neighborhoods are exposed to greater level of stress, and experience poor mental and physical health outcomes [34]. Furthermore, poor neighborhood quality may discourage healthy lifestyle (e.g., physical activity) and increase unhealthy lifestyles (e.g. drinking and smoking)[35]. Moreover, the residents of disadvantaged neighborhood are likely to experience allostatic load (the wear and tear on the body) or chronic activation of the physiological stress response and overexposure to stress hormones, which in turn accelerating telomere shortening [31–33]. Additionally, poor environmental conditions in disadvantaged neighborhood, such as toxic exposures and poor nutrition, can also produce epigenetic modifications [36] and accelerate cellular aging. Conversely, individuals with mental and/or physical disorders are more likely to live in disadvantaged neighborhoods due to functional disability borne from such conditions.

## Materials and Methods

### Study sample

The Netherlands Study of Depression and Anxiety (NESDA) is an ongoing longitudinal cohort study examining the course and consequences of depressive and anxiety disorders. The NESDA sample consists of 2,981 community-residing persons between 18 and 65 years. Participants were recruited between September 2004 and February 2007. The majority (about 95%) of NESDA sample were northern European ancestry. Individuals who had primary clinical diagnosis of severe persistent mental illness (i.e. psychotic disorders, obsessive-compulsive disorder, bipolar disorder, or severe addition disorder) were excluded from the study. Individuals who were not fluent in Dutch language were excluded due to limited funding for translating instruments and for training bilingual interviewers. Also, several instruments were not validated in many languages. Data were collected in four waves: at baseline, 2-, 4-, and 6-year follow-up. The current analyses included baseline data because information on TL was available only at baseline. Baseline assessments included 4-hour face-to-face interview with trained interviewers using computer-assisted modules. In May 2014, to conduct this study, we extracted and analyzed observations collected at baseline with complete data on neighborhood and telomere length (n = 2901). The design and conduct of NESDA have been extensively described in greater detail elsewhere [37]. All participants signed informed consent. The NESDA study was approved by the Institutional Review Board of the following centers: VU University medical center, University Medical Center Groningen and the Leiden University Medical Center.

### Measures

#### Telomere Length (TL)

Leukocyte TL was determined using quantitative polymerase chain reaction (qPCR), adapted from the published original method by Cawthon et al [38] and modified by Lin et al [39]. Telomere sequence copy number in each patient’s sample (T) was compared to a single-copy gene number (S). A single copy gene is a non-telomeric reference sequence that is autosomal (i.e., not a sex chromosome) and non-variable in copy number [40]. In this study, human beta-globin was used as the single-gene copy. The T/S ratio is relative to a reference sample and is proportional to the mean TL.

To estimate T/S ratio, fasting blood was drawn from participants in the morning between 8:30am and 9:30am. DNA samples were stored in a −80°C freezer afterwards. Short specific parts of the template DNA (i.e., telomere sequence and human beta-globin sequence) were amplified using enzyme in cycles. These templates were fluorescently labelled. In every cycle, the number of the template DNAs is doubled, leading to an exponential amplification of target. The amount of the fluorescence released during the amplification is directly proportional to the amount of amplified DNA. We converted T/S ratio into absolute length in base pairs (bp) using the following formulae: bp = 3274-2413x((T/S-0.0545)/1.16). Detailed procedures on collection and storage of blood sample, and extraction and measurement of leukocyte telomere length in NESDA have been fully described elsewhere [13]. TL assaying was conducted at the laboratory of Telome Health Inc. (Menlo Park, CA, USA).

#### Neighborhood Quality

We assessed two dimensions of neighborhood quality: general appraisal of neighborhood and perceived neighborhood quality. General appraisal of neighborhood, adapted from the Social Capital Benchmark Survey [41], was assessed with a question “How would you rate your neighborhood?” We reverse coded the possible answers so that higher scale values indicated more positive appraisal of neighborhood. The possible scores ranged from bad (1), moderate (2), fair (3), good (4), and very good (5).

Perceived neighborhood quality was assessed using three questions. The first two questions were adapted from the scale of perceived neighborhood disorders developed by Ross and Mirowsky [30], and the other was the fear of crime question developed by Baker et al [42]. Each question assessed individual perception of noise, fear of crime and vandalism in the neighborhood: “How often do you experience noise from neighbors, traffic or other sources in your neighborhood (noise)?” “How often do you feel unsafe when you walk alone in your neighborhood (fear of crime)?” and “How often do you see vandalism in your neighborhood such as damaged property (vandalism)?” The possible scores for each question ranged from never [1], seldom [2], sometimes [3], regularly [4], and often [5]. We summed and transformed the values so that the perceived neighborhood quality scores ranged between 0 and 12, using the following formula: perceived neighborhood quality = (noise + fear of crime + vandalism)- 3. Higher scale values indicate greater degrees of negative neighborhood quality (Cronbach’s α = 0.65). In addition to the 12-point scale, we also created a categorical variable in order to visualize findings and check for potential non-linear trends based on the perceived neighborhood quality scores: good neighborhood quality (0–4), moderate neighborhood quality (5–8), and poor neighborhood quality (9–12).

#### Demographic and socioeconomic characteristics

Demographic and socioeconomic characteristics included age (range between18 and 65), gender, currently married/partnered or not, level of education in years, the duration of living in the current address (in years), and poverty status. Poverty status was dichotomized based on monthly household income relative to household size. NESDA collected monthly household income ranging from less than 600 Euros to greater than 5000 Euros, with 200 Euro increment. Individuals living alone (i.e., single households) with monthly income less than 800 Euros per month was coded as living in poverty; all other household composition with monthly household income less than 1200 Euro was coded as living in poverty. This dichotomization was based on the Consensual Budget Study conducted by the Netherlands Institute for Social Research and the National Institute for Family Finance Information [43].

#### Community characteristic

Community characteristic was measured via the zip code based level of urbanization, classified by the Dutch Office for Statistics (CBS) [44]. The level of urbanization is based on the number of persons living per square kilometer: no urbanization [fewer than 500], hardly urbanization [500–1000], moderate urbanization [1000–1500], strong urbanization [1500–2500], and extreme urbanization [more than 2500]).

#### Clinical characteristics

Clinical characteristics included Body Mass Index (underweight, normal weight, over weight and obese), and the number of chronic diseases. We also adjusted for psychiatric indicators, given the nature of this sample and the earlier findings in this study in which depressive and anxiety disorders have been linked to shorter telomeres [13]. We used information on the Inventory of Depression Symptomatology (IDS, range 0–80), the Beck Anxiety Inventory (BAI, range 0–62), and the presence of lifetime major depressive disorder and panic disorder was confirmed using the Diagnostic and Statistical Manual of Mental Disorders 4^th^ Edition (DSM-IV) criteria according to the Composite International Diagnostic Interview (CIDI, version 2.1).

#### Lifestyle characteristics

Lifestyle characteristics included overall energy expenditure in Metabolic Equivalent Total (MET) hours per week [45] and smoking status (never smoked vs. former smoker vs. current smoker). MET is a resting metabolic rate obtained during quiet sitting and used as a method to indicate and compare the absolute intensity and energy expenditure of different physical activities. [46] It is expressed as the oxygen consumption (VO_2_) of approximately 3.5 ml/kg/min [45]. The level of energy consumption associate an activity is expressed multiples of resting MET level. For example, light home activities are 2.3 METs, while competitive bicycling is 16 METs [45]. We calculated MET hours per week using following formula: MET hours per week = (MET level* minutes of activity*number of events per week)/60.

### Statistical Analysis

We first examined the distributions of variables of interest using descriptive statistics. Second, the associations between these variables and TL were examined using linear regression modeling with adjustment for age and gender. This decision was made because it is well documented that age and sex were strongly associated with TL [16, 17]. We also examined the associations between each variable of interest and perceived neighborhood quality scores using unadjusted linear regression modeling. The outcomes of these models were presented in online supplements 1 and 2. Third, to examine the associations between the perceived neighborhood quality and TL, we built five linear regression models with TL as the dependent variable. To examine the effects of potential confounders, we sequentially added a different set of covariates mentioned above, and examined the changes in the magnitude of the associations between TL and each perceived neighborhood quality variable. The first model included age and gender as covariates. Then, we added socioeconomic status, community characteristics, clinical, and life-style related characteristics sequentially. NESDA over sampled individuals with current or lifetime depression. To examine if past or current diagnoses of depression and anxiety disorder modify the associations between perceived neighborhood quality and TL, we added the following interaction terms: each perceived neighborhood quality variable* IDS, each neighborhood quality variable* BAI, each neighborhood quality variable*lifetime major depression, each neighborhood quality variable*lifetime anxiety disorder. Finally, to examine is each sub-domain of perceived quality was associated with TL, we built three linear regression models with each subdomain of perceived neighborhood quality (i.e., noise; fear of crime; and vandalism) as the dependent variable. All analyses were performed with Stata MP version 12.

## Results

The mean TL for the sample was 5472.00 (SD±639.83) base pair (bp). The mean age of sample was 41.90 (SD±13.06). The majority was female (66.4%) and had lifetime diagnosis of depression (64.4%) or anxiety disorder (59.2%)(See Table 1). The mean scores of general appraisal of neighborhood and of perceived neighborhood quality were 3.98 (SD±0.89), and 3.79 (SD±2.32), respectively. The mean scores of noise, fear of crime, and vandalism were 2.78 (SD±1.12), 1.64 (SD±0.84), and 2.37 (SD±1.05), respectively.

**Table 1:** Sample Distribution.

	Mean (±SD)	N (%)
**Telomere Length**	5472.22(±639.84)	
**Neighborhood quality**		
** General appraisal of neighborhood (1–5)**	3.98 (±0.89)	
** Neighborhood Quality (0–12)**	3.79 (±2.32)	
** Good (0–4)**		1,909 (65.78%)
** Medium (5–8)**		892 (30.74%)
** Bad (9–12)**		101 (3.48%)
** Domains of Neighborhood Quality**		
Noise (1–5)	2.78 (±1.12)	
Feel unsafe when walk alone (1–5)	1.64 (±0.84)	
See vandalism (1–5)	2.37 (±1.05)	
**Demographic Characteristics**		
** Age (0–65)**	41.90 (±13.06)	
** Gender**		
Men		973 (33.53%)
Women		1,929 (66.47%)
** North European Ancestry**		
No		146 (5.03%)
Yes		2,756 (94.97%)
** Married/Partnered**		
No		888 (30.6%)
Yes		2,014 (69.4%)
** Living in Poverty**		
No		2,516 (86.7%)
Yes		386 (13.3%)
** Education (Years in school)(5–18 years)**	12.15 (±3.26)	
**Community characteristics**		
** Years of living in the current address**	9.81 (±9.20)	
** Urbanization**		
** <500/m^2^**		1,668 (57.5%)
** 500/m^2^ -1000/m^2^**		374 (12.89%)
** 1000/m^2^-1500/m^2^**		437 (15.06%)
** 1500/m^2^-2500/m^2^**		254 (8.76%)
** >2500/m^2^**		168 (5.79%)
**Clinical Characteristics**		
** Inventory of Depression Symptom score**	21.43 (±14.07)	
** Beck Anxiety Scale**	12.06 (±10.60)	
** Lifetime Major Depression**		1,868 (64.37%)
** Lifetime Anxiety Disorder**		1,717 (59.17%)
** BMI at baseline**	25.61 (±5.00)	
Underweight		64 (2.21%)
Normal weight		1,470 (50.65%)
Overweight		884 (30.46%)
Obese		484 (16.68%)
** Number of Somatic Disease**	0.89 (±1.07)	
**Lifestyle-related characteristics**		
** MET total at baseline (hour/week)**	61.34(±50.54)	
** Smoking**		
Former Smoker		971 (33.46%)
Current Smoker		1,115 (38.42%)
** Heavy drinker**		
No		2,529 (87.13%)
Yes		373 (12.85%)

After adjusting for age and gender, we found that TL was associated with perceived neighborhood quality and each of its domains. On the other hands, we did not find significant association between TL, general appraisal of neighborhood, the number of years living in the current house and the level of urbanization. Inventory of depressive symptom scores and Beck anxiety scale scores were associated with TL and with perceived neighborhood quality (See S1 and S2 Tables).

Table 2 summarizes the multiple multivariable linear regression models with TL as the dependent variable. There were significant linear associations between the perceived neighborhood quality and TL. In the fully adjusted model, a one-point increase in perceived neighborhood quality was associated with approximately 12 bp shortening of TL (b = -12.14, 95% CI: -22.55, -1.72, p = 0.022). In other words, compared to those reporting good neighborhood quality, those who reported moderate neighborhood quality had approximately 69 bp shorter TL (b = -69.33, 95% CI: -119.49, -19.17, p = 0.007). We did not find any significant interaction term mentioned in the previous section.

**Table 2:** Associations between Telomere Length and Perceived Neighborhood Quality.

	Model I			Model II			Model III			Model IV		
			N = 2902; R^2^ = 0.10			N = 2901; R^2^ = 0.10			N = 2895; R^2^ = 0.10			N = 2895; R^2^ = 0.11
	β	P>|t|	[95% CI]	β	P>|t|	[95% CI]	β	P>|t|	[95% CI]	β	P>|t|	[95% CI]
**Perceived Neighborhood quality**												
Good	**Ref**			**Ref**			**Ref**			**Ref**		
Poor	-84.42	<0.001	[-132.87, -35.98]	-82.46	<0.001	[-132.05,-32.88]	-73.71	0.004	[-123.81,-23.61]	-69.33	0.007	[-119.49,-19.17]
Bad	-193.03	<0.001	[-314.73, -71.33]	-190.34	<0.002	[-313.40,-57.28]	-181.19	0.004	[-306.07,-56.31]	-173.80	0.006	[-298.60,-49.01]

**Model I:** Adjusted for age, gender, and demographic characteristics.

**Model II:** Adjusted for age, gender, demographic, and community characteristics.

**Model III:** Adjusted for age, gender, demographic, community, and clinical characteristics.

**Model IV:** Adjusted for age, gender, demographic, community, clinical, and lifestyle characteristics.

**Fig 2:**
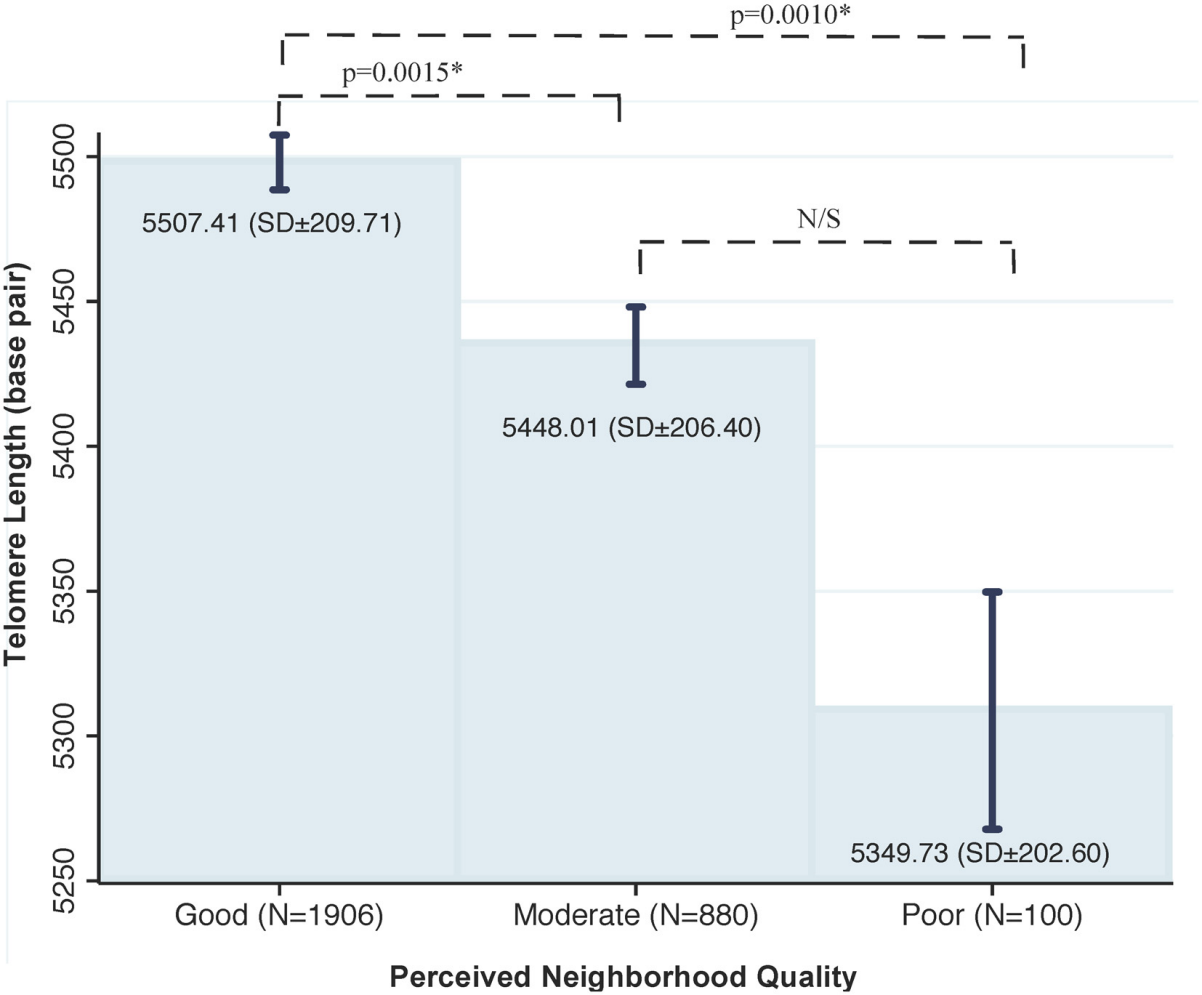
The Association between Telomere Length and Perceived Neighborhood Quality. After adjusting for a comprehensive set of covariates, those living in greater unfavorable neighborhoods quality had shorter telomere length.

The difference in mean TL between those reporting good neighborhood quality and poor neighborhood quality was about 174 bp (b = -173.80, 95% CI: -298.80,- 49.01 p = 0.006). The difference in mean TL between those reporting moderate and poor neighborhood quality was not statistically significant (p = 0.111). Fig 2 presents mean TL and 95% CIs for the three groups based on the neighborhood quality scale after adjusting for full set of covariates.

## Discussion

Our primary hypothesis was that poor neighborhood quality would be associated with shortened TL. We found that overall index scores of perceived neighborhood quality were significantly associated with accelerated cellular aging even after adjusting for comprehensive sets of covariates: demographic, socioeconomic, community, clinical, and lifestyle characteristics. We also observed a dose response of poor perceived neighborhood quality to cellular aging; the greater degrees of unfavorable perceived neighborhood quality, the shorter the mean TL was. Based on an estimated mean telomere shortening rate of 14–20 bp per year as found in this and other studies [47], the differences observed indicate 8.7–11.9 years of accelerated cellular aging for individuals reporting poor neighborhood quality as compared to those reporting good neighborhood quality. On the other hand, we did not find significant associations between general appraisal of neighborhood and TL. This may be due to that fact that the majority of sample (n = 2,248, 77.5%) rated their neighborhood very good or good, while only 149 respondents (6.65%) rated their neighborhood moderate or bad. Such high degrees of satisfaction with neighborhood in overall sample may have lowered statistical power to detect meaningful differences in TL between varying degrees of general appraisal of neighborhood.

Our secondary hypothesis was that each sub-domain of perceived neighborhood quality would be independently associated with TL. We found that, while fear of crime and vandalism were significantly associated with TL, noise was not associated with the TL after adjusting for age, gender, and demographic characteristics. This finding seems to suggest that different neighborhood characteristics have differential impacts on cellular aging. In other words, unfavorable emotional milieu, such as living in fear of crime, may have more direct impact on cellular aging than the high levels of perceived noise.

To our knowledge, this is one of three studies examining the associations between perceived neighborhood quality and cellular aging. Although each study used a unique study sample and measurements, these studies collectively establish the association between TL and the neighborhood quality perceived by residents. Theall et al [48] examined salivary TL in a sample of 99 children age between 4 and 14 recruited from urban New Orleans, Louisiana. The authors concluded that children living in disadvantaged neighborhoods (greater level of neighborhood disorder by the mother’s appraisal) had lower salivary TL than those who did not. Needham et al [49] examined the associations between several neighborhood characteristics and leukocyte TL, using multi-ethnic U.S. sample aged between 45 and 84. The authors concluded that individuals who lived in neighborhood with lower aesthetic quality, safety, and social cohesion had shortened TL than those who lived in neighborhoods with a more favorable social environment, after controlling for individual-level socioeconomic status, neighborhood socioeconomic disadvantage, and biomedical and lifestyle risk factors. In all three studies, interestingly, the authors did not find significant associations between TL and objective measures of neighborhood quality: neighborhood-level economic deprivation (Theall et al), neighborhood SES (Neehan et al), and population density (current study). More studies are needed to examine why subjective indicators, but not objective indicators, of neighborhood quality are associated with TL.

A unique contribution of our study is that we extensively examined effects of depression and anxiety in the association between perceived neighborhood quality and TL. Having depression and anxiety may “paint” one’s surroundings more negative tones. Thus, the association between the perceived neighborhood quality and TL found in the literature could have been a reflection of one’s emotional status. However, we found that the association between perceived neighborhood quality and TL remained significant even after adjusting for four measures of depression and anxiety (i.e., depressive symptoms, anxiety symptoms, lifetime diagnoses of depression and of anxiety disorders). Furthermore, we did not find significantly modifying effects of depression and anxiety in such association. Our finding suggests that the relation between the perceived neighborhood quality and TL is not entirely a reflection of one’s emotional status. Moreover, our finding may be confirmative of the general notion that perceived neighborhood quality is indicative of objective neighborhood quality. Existing literature has found a good correlation between objective and subjective measures of neighborhood quality in general population [50–52]. For example, individuals living in high crime areas report high levels of fear of crime [53–55]. Our study suggests that these may be also true in the sample including a large number of individuals with depression and anxiety disorders.

There are few limitations in this study. First, due to the cross-sectional nature of this study, we are unable to make causal inferences about the relations between TL and perceived neighborhood quality. Second, although time at current residence was controlled for, we were not able to examine if and how changes in neighborhood quality over time impacted TL. Third, due to the sample characteristics, generalizability of our findings to older adults and to racially diverse populations may be limited. The NESDA baseline sample does not include individuals older than 65. Also, only a small number of participants (n = 146; 5.03%) identified themselves as non-northern European ancestries. Fourth, psychometric property of the perceived neighborhood quality (Cronbach’s alpha = 0.65) is not very strong. This may be due to the fact it comprise only three items. Also, the participants generally rated their neighborhood favorably with relatively small variations. For example, the mean score of fear of crime was 1.64 (SD±0.84). This may have lowered our statistical power.

Despite above limitations, our study is an important addition to growing body of literature on factors associated with cellular aging. In this study we have established that certain neighborhood characteristics matter for cellular aging over and above a range of individual attributes. The findings of this study are indicative of potential pathways that have not been previously examined and offer new directions for measurement and research on cellular aging. Furthermore, biological aging studies that focus purely on individual characteristics may have limited power to explain important correlates and determinants of aging and of health. This study has shown the importance of integrating research on social processes related to health and cellular aging.

## Supporting Information

S1. Table Associations between Variables of Interest and Telomere Length. (DOCX)

S2. Table Associations between Variables of Interest and Perceived Neighborhood Quality. (DOCX)
