# Supplementary material for: Where You Live May Make You Old: The Association between Perceived Poor Neighborhood Quality and Leukocyte Telomere Length
Source: PLoS One. 2015 Jun 17;10(6):e0128460. doi: 10.1371/journal.pone.0128460 (PMC4471265; doi:10.1371/journal.pone.0128460)
Supplement: S1. Table — (DOCX) [file pone.0128460.s001.docx]

| **S1 Table: Associations between Variables of Interest and Telomere Length^1^** | | | | | |
| --- | --- | --- | --- | --- | --- |
|  | | Coef. | | 95% CI | |
| **General Appraisal of Neighborhood (0-5)** | | 2.27 | | [-22.73, 27.27] | |
| **Perceived Neighborhood Quality (0-12)** | | -16.99*** | | [-26.57, -7.416] | |
| **Perceived Neighborhood Quality (3 categories)** | |  | |  | |
| Good | | Ref | |  | |
| Medium | | -84.42*** | | [-132.9, -35.98] | |
| Bad | | -193.0** | | [-314.7, -71.33] | |
| **Domains of Neighborhood Quality** | |  | |  | |
| Noise (1-5) | | -24.54* | | [-44.45, -4.63] | |
| Feel unsafe when walk alone (1-5) | | -40.90** | | [-67.75, -14.04] | |
| See vandalism (1-5) | | -31.16** | | [-52.21, -10.11] | |
| **Demographic Characteristics** | |  | |  | |
| Married/Partnered | | 3.89** | | [-44.51, 52.29] | |
| North European Ancestry | | 146.62** | | [45.26, 247.98] | |
| Living in poverty | | -32.82 | | [-86.71, 21.08] | |
| Education (Years in school)(5~18 years) | | 0.918 | | [-5.89, 7.72] | |
| Years of living in the current address | | 1.42 | | [-1.34, 4.19] | |
| **Community characteristics** | |  | |  | |
| Urbanization (1-5) | | 14.8 | | [-2.92, 32.53] | |
| **Clinical Characteristics** | |  | |  | |
| Inventory of Depression Symptom score | | -2.481** | | [-4.06, -0.90] | |
| Beck Anxiety Scale | | -2.85** | | [-4.94, -0.75] | |
| Lifetime Major Depression | | -32.19 | | [-78.58, 14.20] | |
| Lifetime Anxiety Disorder | | -54.26* | | [-99.44, -9.09] | |
| BMI Categories | |  | |  | |
| Underweight | | 119.3 | | [-34.08, 272.7] | |
| Normal weight | | Ref | | Ref | |
| Overweight | | -55.72* | | [-108.1, -3.35] | |
| Obese | | -44.35 | | [-108.1, 19.45] | |
| Number of Somatic Disease | | 1.54 | | [-20.39, 23.48] | |
| **Lifestyle-related characteristics** | |  | |  | |
| MET total at baseline (hour/week) | | -0.21 | | [-0.65, 0.23] | |
| Smoking | |  | |  | |
| Never Smoked | | Ref | |  | |
| Former Smoker | | -30.69 | | [-88.52, 27.14] | |
| Current Smoker | | -92.14** | | [-147.2, -37.06] | |
| Heavy drinker | | -66.68 | | [-133.25, -0.11] | |
| **^1^Adjusted for age and gender**  * p<0.05, ** p<0.01, *** p<0.001 | | | | | |
