# Supplementary material for: Where You Live May Make You Old: The Association between Perceived Poor Neighborhood Quality and Leukocyte Telomere Length
Source: PLoS One. 2015 Jun 17;10(6):e0128460. doi: 10.1371/journal.pone.0128460 (PMC4471265; doi:10.1371/journal.pone.0128460)
Supplement: S2. Table — (DOCX) [file pone.0128460.s002.docx]

| **S2 Table: Associations between Variables of Interest and Perceived Neighborhood Quality** | | |  |
| --- | --- | --- | --- |
|  | | Coef. | 95% CI |
| **Demographic Characteristics** | |  |  |
| Age | | -0.005 | [-0.01, 0.002] |
| Woman (ref: men) | | 0.48*** | [0.39, 0.65] |
| North European Ancestry | | -0.07 | [-0.46, 0.32] |
| Married/Partnered | | -0.62*** | [-0.80, -0.44] |
| Living in poverty | | 0.96*** | [0.76, 1.16] |
| Education (Years in school)(5~18 years) | | 0.01 | [-0.02, 0.04] |
| Years of living in the current address | | -0.01 | [-0.01, 0.004] |
| **Community characteristics** | |  |  |
| Urbanization (1-5) | | -0.44*** | [-0.50, -0.37] |
| **Clinical Characteristics** | |  |  |
| Inventory of Depression Symptom score | | 0.03*** | [0.02, 0.036] |
| Beck Anxiety Scale | | 0.04*** | [0.03, 0.05] |
| Lifetime Major Depression | | 0.37*** | [0.19, 0.54] |
| Lifetime Anxiety Disorder | | 0.55*** | [0.38, 0.72] |
| BMI Categories | |  |  |
| Underweight | | -0.01 | [-0.59, -0.57] |
| Normal weight | | Ref | Ref |
| Overweight | | -0.19 | [-0.39, 0.0001] |
| Obese | | -0.12 | [-0.36, 0.12] |
| Number of Somatic Disease | | 0.14*** | [0.06, 0.22] |
| **Lifestyle-related characteristics** | |  |  |
| MET total at baseline (hour/week) | | 0.003*** | [0.001, 0.00] |
| Smoking | |  |  |
| Never Smoked | | Ref | Ref |
| Former Smoker | | 0.26* | [0.05, 0.47] |
| Current Smoker | | 0.23 | [-0.03, 0.48] |
| Heavy drinker | | 0.003*** | [0.00, 0.005] |
| * p<0.05, ** p<0.01, *** p<0.001 | | | |
